# Supplementary material for: Network Pharmacology and Experimental Validation Identify Paeoniflorin as a Novel SRC-Targeted Therapy for Castration-Resistant Prostate Cancer
Source: Pharmaceuticals (Basel). 2025 Aug 21;18(8):1241. doi: 10.3390/ph18081241 (PMC12388997; doi:10.3390/ph18081241)
Supplement: Supplementary file 1 [file pharmaceuticals-18-01241-s001.zip › pharmaceuticals-3778000-supplementary.pdf]

Supplementary Table S1. Primers sequence used in the study

RT-qPCR (5'-3')		
GAPDH	Forward	CTGGGCTACACTGAGCACC
	Reverse	AAGTGGTCGTTGAGGGCAATG
SRC	Forward	GAGCGGCTCCAGATTGTCAA
	Reverse	CTGGGGATGTAGCCTGTCTGT
TP53	Forward	CAGCACATGACGGAGGTTGT
	Reverse	TCATCCAAATACTCCACACGC
EGFR	Forward	AGGCACGAGTAACAAGCTCAC
	Reverse	ATGAGGACATAACCAGCCACC
AKT1	Forward	AGCGACGTGGCTATTGTGAAG
	Reverse	GCCATCATTCTTGAGGAGGAAGT
AR	Forward	GTGGAAGCTGCAAGGTCTTC
	Reverse	TTCAGATTACCAAGTTTCTTCAGC
ESR1	Forward	CCCACTCAACAGCGTGTCTC
	Reverse	CGTCGATTATCTGAATTTGGCCT
TGFBR2	Forward	GTAGCTCTGATGAGTGCAATGAC
	Reverse	CAGATATGGCAACTCCCAGTG
NQO1	Forward	GAAGAGCACTGATCGTACTGGC
	Reverse	GGATACTGAAAGTTCGCAGGG
ALDH2	Forward	ATGGCAAGCCCTATGTCATCT
	Reverse	CCGTGGTACTTATCAGCCCA
ADH1B	Forward	CCCGGAGAGCAACTACTGC
	Reverse	AACCAGTCGAGAATCCACAGC
